# Risk of Injury in Moral Dilemmas With Autonomous Vehicles

**DOI:** 10.3389/frobt.2020.572529

**Published:** 2021-01-20

**Authors:** Celso M. de Melo, Stacy Marsella, Jonathan Gratch

**Affiliations:** ^1^ CCDC US Army Research Laboratory, Playa Vista, CA, United States; ^2^ College of Computer and Information Science, Northeastern University, Boston, MA, United States; ^3^ Institute for Creative Technologies, University of Southern, Playa Vista, CA, United States

**Keywords:** ethics, utilitarian choice, automated vehicles, moral dilemma, risk of injury

## Abstract

As autonomous machines, such as automated vehicles (AVs) and robots, become pervasive in society, they will inevitably face moral dilemmas where they must make decisions that risk injuring humans. However, prior research has framed these dilemmas in starkly simple terms, i.e., framing decisions as life and death and neglecting the influence of risk of injury to the involved parties on the outcome. Here, we focus on this gap and present experimental work that systematically studies the effect of risk of injury on the decisions people make in these dilemmas. In four experiments, participants were asked to program their AVs to either save five pedestrians, which we refer to as the utilitarian choice, or save the driver, which we refer to as the nonutilitarian choice. The results indicate that most participants made the utilitarian choice but that this choice was moderated in important ways by perceived risk to the driver and risk to the pedestrians. As a second contribution, we demonstrate the value of formulating AV moral dilemmas in a game-theoretic framework that considers the possible influence of others’ behavior. In the fourth experiment, we show that participants were more (less) likely to make the utilitarian choice, the more utilitarian (nonutilitarian) other drivers behaved; furthermore, unlike the game-theoretic prediction that decision-makers inevitably converge to nonutilitarianism, we found significant evidence of utilitarianism. We discuss theoretical implications for our understanding of human decision-making in moral dilemmas and practical guidelines for the design of autonomous machines that solve these dilemmas while, at the same time, being likely to be adopted in practice.

## 1 Introduction

As autonomous machines—robots, drones, self-driving cars, etc.—quickly become a reality, they are bound to face moral dilemmas where a decision must be made between two or more negative outcomes (Deng, 2015). Given the increasing amounts of investment and promising results (Waldrop, 2015), studying these dilemmas is particularly important in the domain of automated vehicles (AVs) (Lin, 2015). Imagine that an AV is driving through a tunnel and is suddenly faced with several pedestrians crossing in the middle of the road; should the AV swerve against the wall—injuring or even killing the driver—or continue moving forward—injuring or even killing the pedestrians? Prior research suggests that in this situation, many people make the choice that saves the most lives and, in this case, possibly results in the driver’s death to be the appropriate choice (Bonnefon et al., 2016; Awad et al., 2018; Faulhaber et al., 2019; McManus and Rutchick, 2019). However, paradoxically, people also report a preference to purchase for themselves an AV that prioritizes the safety of the driver (Bonnefon et al., 2016). This dilemma, thus, highlights the tradeoff that must be made between machines that try to maximize collective welfare and machines that people will actually adopt. It is, therefore, critical that we understand the factors that shape people’s decision-making in such dilemmas. Here, we focus on one factor that has been mostly ignored in the experimental literature on moral dilemmas involving AVs—*risk of injury to the involved parties*—and demonstrate its critical importance to people's decision-making. As another contribution, we present experimental evidence that these decisions are also shaped by how others, facing a similar situation, decide, which emphasizes important social considerations often missing in prior work.

### 1.1 What Is the Moral Choice?

First, however, it is important to clarify what is meant by the moral choice in these moral dilemmas. This question has been debated for centuries by philosophers (Kant, 1797; Bentham, 1879; Mill, 1863; Scanlon, 1998; Singer, 2017) but here, though, we try to avoid adopting any position on what the moral choice should be. Instead, consistent with prior terminology (Faulhaber et al., 2019), we simply refer to the two possible choices as *utilitarian*, which minimizes the number of individuals facing injury or death and, in our case, spares the pedestrians, and *nonutilitarian*, which protects the health of the individual and, in our case, spares the driver.^1^ We, thus, aim to avoid some of the complications introduced by various consequentialist and deontological views of moral choice; in particular, we consider a random choice or refusal to make a choice as being out of scope for this work.

### 1.2 Risk of Injury vs. Certain Death

Prior research has mostly focused on moral dilemmas where the outcomes lead to the death of the targeted human(s) with 100% certainty (Bonnefon et al., 2016; Faulhaber et al., 2019; McManus and Rutchick, 2019). Whereas these extreme cases are important to understand people’s moral decisions, in reality, many outcomes are unlikely to lead to certain death. This is an important distinction as different risk profiles can influence people’s moral decision-making (Goodall, 2016). In fact, Bonnefon et al. (2016) note in their discussion that “a collective discussion about moral algorithms will have to tackle the concepts of expected risk” (pg. 1,576). Faulhaber et al. (2019) also point that “Although phrasing the (…) dilemma not in terms of life and death but in terms of health or injury is equivalent, it might lead to differences in decision-making” (pg. 408); moreover, in one experiment, they show that participants preferred to hit a standing adult than a kneeling adult, thus revealing an implicit concern for risk of injury. While advocating for more sophisticated decision-analytic reasoning than what is prescribed by moral rules (Bennis et al., 2010), Bazerman and Greene (2010) also note the importance of accounting for probabilistic outcomes when solving moral dilemmas. Nevertheless, a comprehensive study of the influence of risk of injury, on the self and others, on people's decisions in moral dilemmas involving AVs is still missing and here we address this important gap. Based on the comments above, we advance two general hypotheses: (H1) people will be less likely to make the utilitarian choice, the higher the risk to the driver; and (H2) people will be more likely to make the utilitarian choice, the higher the risk to the pedestrians. One research question, though, is (RQ1) how will the risk to driver interact with the risk to pedestrians to shape the utilitarian choice?

### 1.3 Experimental Methodology

Prior research has mostly followed an experimental approach based on human subject studies to gauge people's moral decisions. To avoid the ethical difficulties of running experiments about life and death situations, most of the earlier work asked participants to make a moral choice or judgment in the context of hypothetical (Greene et al., 2001; Mikhail, 2007) or presumably real (Rutchick et al., 2017; Bostyn et al., 2018) moral dilemma scenarios. In the context of self-driving cars, prior work has studied participants' choices in hypothetical scenarios (Awad et al., 2018; McManus and Rutchick, 2019) and virtual reality simulations (Pan and Slater, 2011; Francis et al., 2016; Faulhaber et al., 2019). Following this tradition, in this paper, we present several experiments where we manipulate the likelihood of injury to drivers and pedestrians in a moral dilemma scenario involving AVs and measure participants’ decision-making.

### 1.4 Game-Theoretic Formulation

**FIGURE 1:**
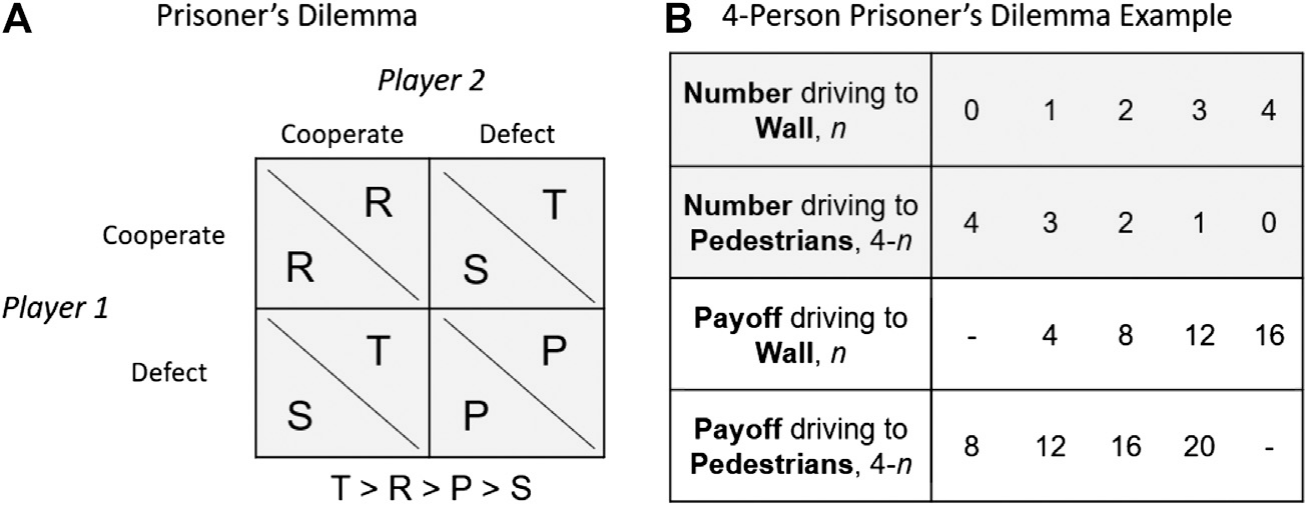
Game-theoretic formulation for moral dilemmas involving AVs: **(A)** the prisoner’s dilemma; **(B)** an example of a formulation of the AV moral dilemma as a 4-person prisoner’s dilemma.

Conitzer et al. (2017) argued (behavioral) game theory is an appropriate framework to model moral decision-making in artificial intelligence. Here, we also follow a game-theoretic formulation based on *social dilemmas*, which are situations that capture a conflict between individual and collective interests (Dawes, 1980; Kollock, 1998). Specifically, consider the *prisoner's dilemma*, which is a social dilemma involving two individuals that have to make a simultaneous decision to either cooperate or defect (see Figure 1A). If they both cooperate, they each receive a payoff *R*. If they both defect, they receive a payoff *P* that is lower than *R*. However, if one cooperates and the other defects, the defector earns the highest possible reward (*T*) and the cooperator the lowest (*S*), i.e., T>R>P>S. Rational theory predicts that, in this case, both players should defect (von Neumann and Morgenstern, 1944): if one believes the other will cooperate, then the greater payoff is to defect; however, if all players think like this, then they will achieve mutual defection, which is of course worse than mutual cooperation.

Gogoll and Müller (2017) argued that moral dilemmas involving AVs are prisoner's dilemmas involving multiple owners of AVs. To make it concrete, imagine that an individual must program his/her AV simultaneously as three other owners. Imagine further that we consider the utilitarian choice—programming to risk the driver, thus sparing the five pedestrians—to be cooperation and the nonutilitarian choice to be defection, and we define a payoff matrix as shown in Figure 1B. This is similar to the formulation advanced by de Melo et al. (2019) in the context of a nonmoral dilemma that did not involve (direct) risk to human life. If the individual chooses to drive towards the wall and the remaining players choose to drive towards the pedestrians, then the individual gets four points, whereas the other players get 12 points each (this is the second column in the payoff table); this is the worst possible outcome for the individual. If everyone decides to drive towards the pedestrians, then everyone gets only eight points (first column); this is mutual defection. In contrast, if everybody chooses to drive towards the wall, then everybody gets 16 points (fifth column); this is the best outcome for the collective, i.e., mutual cooperation. As noted previously, the rational prediction is that all owners should choose to defect and, thus, all would program to drive towards the pedestrians. For this reason, Gogoll and Müller (2017) propose that owners should *not* be given the opportunity to program their AVs and an external institution should impose the utilitarian choice on AV owners.

However, several decades of experimental work have accumulated considerable evidence that people will often not follow the rational prediction and, in fact, cooperate in the prisoner's dilemma (Dawes, 1980; Kollock, 1998; Rand and Nowak, 2013). In dilemmas not involving AVs, research indicates that moral decision often conformed to the actions of the majority (Crutchfield, 1955; Hornsey et al., 2003; Kundu and Cummins, 2013; Bostyn and Roets, 2017) and was subject to strategic concerns about how one was perceived by others (Rom and Conway, 2018). Moreover, Gogoll and Müller (2017) present a theoretical argument and do not advance experimental evidence that 1) individuals' decisions are influenced by what others decide and 2) individuals do, in fact, always choose to “defect.” Here we formulate the moral dilemma as a 4-person social dilemma to allow for these types of social influence to occur and, furthermore, in our last experiment, test the hypothesis that participants will be influenced by the decisions made by others (H3). If true, this would suggest that framing moral dilemmas as social group dilemmas may be more ecologically valid. However, given prior work on cooperation in the prisoner’s dilemma and on moral conformity, contrasting to Gogoll and Müller’s prediction, we hypothesize that participants will not strategically converge to the nonutilitarian choice (H4).

In our game-theoretic formulation, though, we do not associate an explicit payoff matrix (such as the one exemplified in Figure 1B) with the moral dilemmas. Whereas experimental economists often insist that payoff matrices and corresponding financial incentives be clearly defined for any experimental game (Hertwig and Ortmann, 2001), there is some evidence that people can behave differently in situations involving moral values and that it may be inappropriate to associate monetary rewards to decisions involving human life (Tetlock, 2003; Dehghani et al., 2014; Wang et al., 2018). It is not clear, thus, if associating a formal payoff matrix would be detrimental to the study of moral dilemmas with AVs and, therefore, we chose to simply avoid the topic and leave it for future work (but see the Supplemental Material for some preliminary work on financial incentives).

### 1.5 Approach and Contributions

We present three experiments that systematically study the impact of risk of injury on participants' decision-making and a fourth experiment that studies social influence in moral dilemmas involving AVs. In our first experiment, we study decision-making in scenarios where the risk of injury is the same for the driver and (five) pedestrians. This experiment confirms that participants’ decisions are influenced by risk. Our second experiment, then, studies decision-making in more ecologically valid scenarios where the risk of injury to the driver is not necessarily the same as for pedestrians. This experiment reinforces the influence of risk and reveals that the participants’ willingness to make the utilitarian choice depends not only on how low is the risk to the driver but also on how high is the risk to the pedestrians. The third experiment seeks to identify the exact decision function for the utilitarian choice by asking participants to report the threshold for pedestrians' risk at which they would switch to the utilitarian choice, at different levels of risk to the driver. The results indicate a linear function, where people require higher risk to pedestrians, the higher the risk to the driver; however, the slope of this function is lower than one, thus suggesting that participants will make the utilitarian choice even if risk to pedestrians is not as high as to the driver. Finally, our fourth experiment matches participants with other drivers that tend to make either the utilitarian or nonutilitarian choice. The results indicate that others’ behavior influences participants’ decisions, with participants tending to make the utilitarian choice the more others are to do the same.

## 2 Method

### 2.1 Experiment 1

In this experiment, participants engaged in a 4-person prisoner’s dilemma scenario where they had to choose between swerving towards a wall (utilitarian choice) or continuing forward towards five pedestrians (nonutilitarian choice). Participants were instructed that they would program their AV to act on their behalf and that they would not learn about the other participants’ decisions until after the scenario was over. Participants were quizzed on all instructions prior to starting the task and they were not allowed to proceed until they successfully completed the quiz.

The experiment followed a repeated-measures design with five levels^2^: risk of injury (to drivers and pedestrians), 10% vs. 30% vs. 50% vs. 70% vs. 90%. Participants were instructed that their decisions would lead to a “chance of serious injury for yourself/pedestrians (including death)” corresponding to a certain risk probability defined by the experimental condition. The risk of injury was equivalent for driver and pedestrians in each condition. This factor was within-subjects, meaning that participants reported their decisions for each risk of injury probability. The order for these conditions was counterbalanced across participants.

The experiment was fully anonymous for participants. To accomplish this, counterparts were referred to as “anonymous” and we never collected any information that could identify participants. To preserve anonymity with respect to experimenters, we relied on the anonymity system of the online pool we used, Amazon Mechanical Turk. When interacting with participants, researchers are never able to identify participants, unless we explicitly ask for information that may serve to identify them (e.g., name or photo), which we did not. This experimental procedure is meant to minimize any possible reputation effects, such as a concern for future retaliation for the decisions made in the scenarios.

**FIGURE 2:**
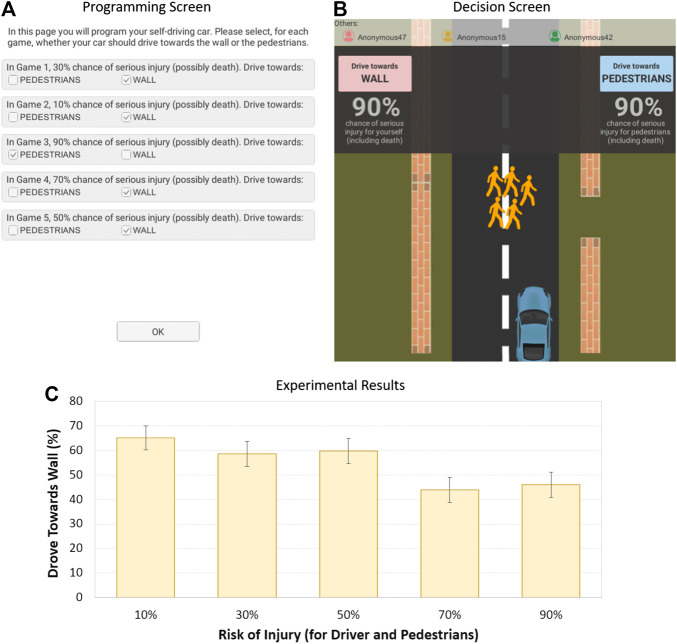
Moral dilemma software and results in Experiment 1: **(A)** programming interface; **(B)** decision screen; **(C)** the influence of risk of injury on utilitarian choice (error bars show standard errors).

After programming the AV, participants would see a simulation where the AV would be driving down a road and, as it entered a tunnel, five pedestrians would suddenly cross the road at which point the simulation would stop for a few seconds showing the available options and risk of injury (Figure 2B). However, since the decisions were programmed, the simulation would automatically resume and execute the programmed decision for the corresponding risk of injury. The simulation would then show the car hitting either the wall or pedestrians (no graphic imagery or sounds were performed). See the Supplemental Material for a video of the experimental software.

After each scenario, the participant would learn the decisions made by their counterparts. The counterpart decisions were predefined and followed a pattern that was neither too cooperative nor competitive: Wall-Wall-Pedestrians (Scenario 1), Pedestrians-Pedestrians-Wall, Wall-Pedestrians-Wall, Pedestrians-Wall-Pedestrians, and Pedestrians-Wall-Wall (Scenario 5). Before starting the next scenario, participants were given the opportunity to reprogram their AV for the remaining scenarios.

Before starting the task, participants had to wait for approximately 30 s while “they waited for other participants to join.” However, in order to increase experimental control, participants always engaged with a computer script that simulated the other participants. Similar experimental manipulations have been used in other experiments studying behavior involving intelligent machines (e.g., de Melo et al. (2019)). Participants were fully debriefed about this experimental procedure at the end of the experiment. All the experimental methods used in the experiment were approved by the University of Southern California IRB (ID UP-14–00,177) and the US Army Research Lab IRB (ID ARL 18–002).

All participants were recruited from Amazon Mechanical Turk. Previous research shows that studies performed on Mechanical Turk can yield high-quality data and successfully replicate the results of behavioral studies performed on traditional pools (Chandler and Ipeirotis, 2010). In every experiment, we only sampled participants from the United States with an excellent performance history (95% approval rate on previous Mechanical Turk tasks). We recruited 94 participants—see Supplemental Material for details—for all experiments, on sample size calculation, sample demographics, and financial incentives. To analyze the data, we ran a repeated measure analysis of variance (ANOVA) on the utilitarian choice rate (i.e., the average number of times participants chose to swerve towards the wall). The results revealed a main effect of risk of injury, *F* (4, 372) = 4.544, *p* = 0.001, and $\eta_{p}^{2}$ = 0.047, with people being less likely to swerve towards the wall, the higher the risk of injury to the driver and pedestrians; see Figure 2C and detailed statistics in the Supplemental Material. This result confirms that the risk of injury influences people’s likelihood of making the utilitarian choice. But, how do risk to driver and risk to pedestrians separately influence decision-making? To get insight, we followed up with an experiment where the risks to the driver and pedestrians varied independently.

### 2.2 Experiment 2

**FIGURE 3:**
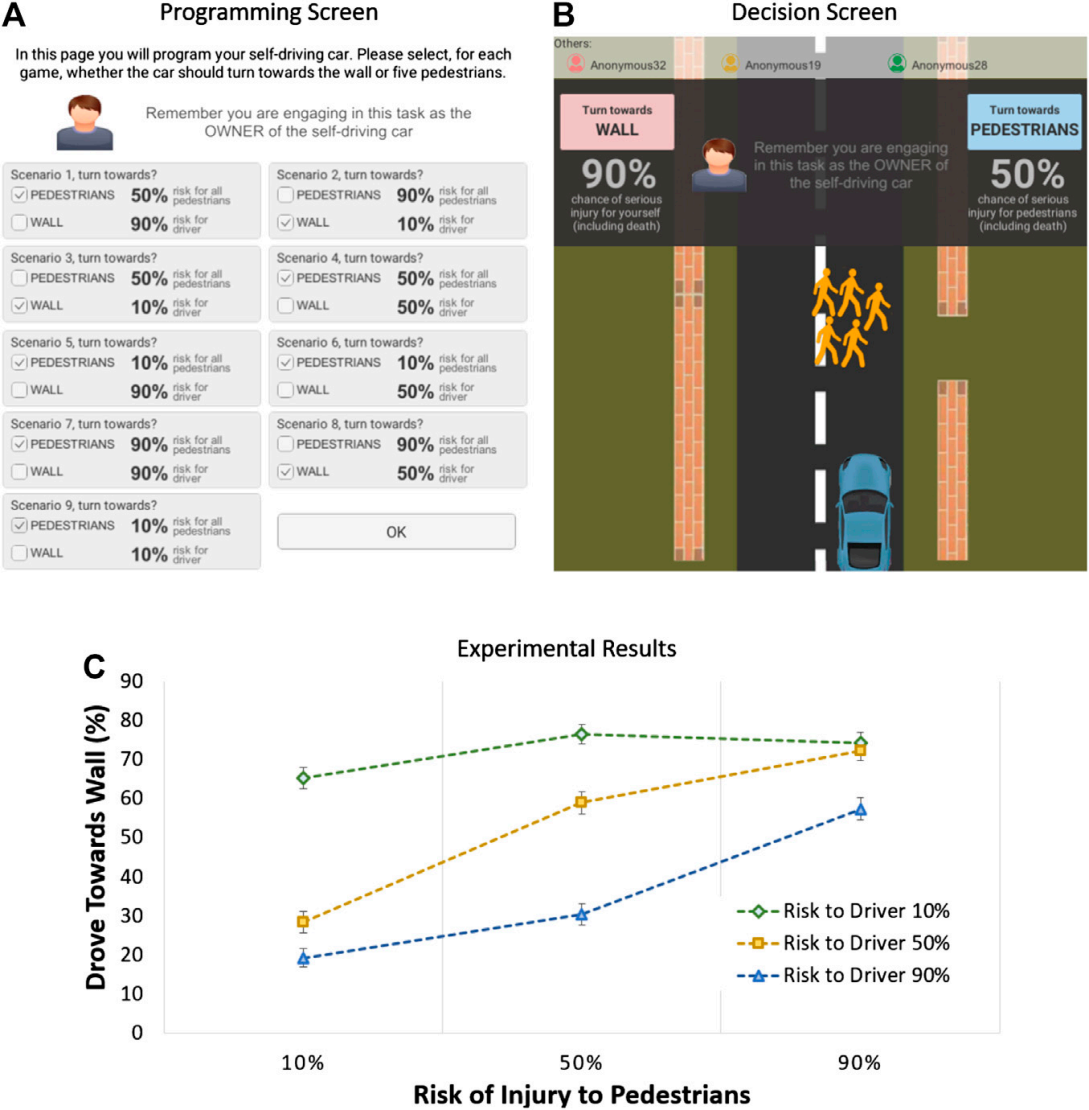
Moral dilemma software and results in Experiment 2: **(A)** programming interface; **(B)** decision screen; **(C)** the influence of risk of injury on utilitarian choice (error bars show standard errors).

The second experiment extended the previous one in one important way: we studied mixed risk profiles, where the risk of injury for the pedestrians was not necessarily the same as for the driver. The experiment followed a repeated measure 3 × 3 design^3^: risk of injury to driver (10% vs. 50% vs. 90%) × risk of injury to pedestrians (10% vs. 50% vs. 90%). The (4-person) moral dilemma was the same as in Experiment 1, except that participants engaged, in this case, in nine scenarios corresponding to all possible combinations of risk of injury for driver and pedestrians. The order for these scenarios was counterbalanced across participants. Figures 3A and B show screenshots of the software.

We recruited a sample of 276 participants from Amazon Mechanical Turk. This was a new sample and we always made sure to avoid repeating participants across experiments. To analyze the data, we ran a risk of injury to driver × risk of injury to pedestrian repeated measure ANOVA on the utilitarian choice rate. The results, shown in Figure 3C and detailed in the Supplemental Material, revealed the main effect of risk to the driver, *F* (2, 1,124) = 140.09, *p* < .001, and $\eta_{p}^{2}$ = 0.333: people were more likely to make the utilitarian choice, the lower the risk for the driver; this, thus, confirmed our hypothesis H1. The results also showed the main effect of risk on pedestrians, *F* (2, 1,124) = 106.82, *p* < .001, and $\eta_{p}^{2}$ = 0.275: people were more likely to make the utilitarian choice, the higher the risk to pedestrians; this confirmed our hypothesis H2. Interestingly, there was a statistically significant risk to driver × risk to pedestrian interaction, *F* (4, 1,124) = 23.01, *p* < .001, and $\eta_{p}^{2}$ = 0.076: when the risk to the driver was low, participants tended to make the utilitarian choice; however, when the risk to the driver was medium or high, the likelihood of making the utilitarian choice decreased with risk to the driver. The results from the experiment confirm that decision-makers were influenced by the combined effect of risk to driver and risk to pedestrians. The next experiment sought to get further insight on this decision function and, in particular, understand when participants switch from the nonutilitarian to the utilitarian choice.

### 2.3 Experiment 3

**FIGURE 4:**
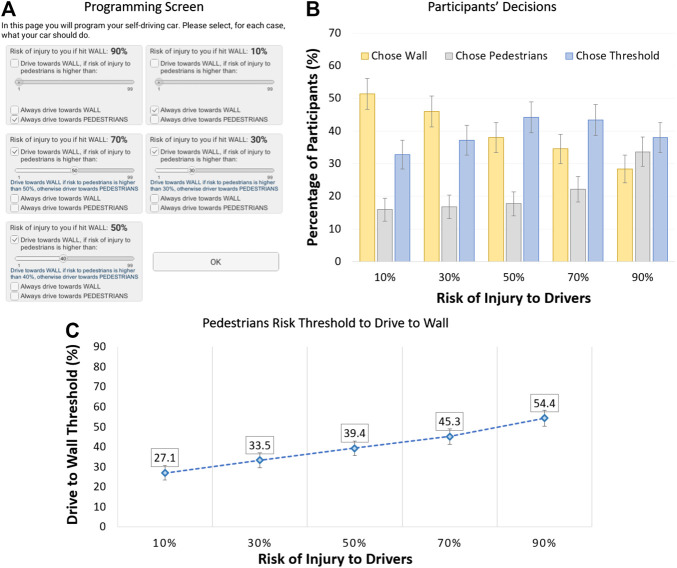
Moral dilemma software and results in Experiment 3: **(A)** programming interface; **(B)** percentage of participants that always chose wall, always chose pedestrians, or chose to specify a switch threshold; **(C)** threshold for pedestrians risk at which participants switch to utilitarian choice, for various levels of risk to the driver (error bars show standard errors).

Risk to driver and risk to pedestrians define a bidimensional decision space. At each point in this space, a decision needs to be made, utilitarian vs. nonutilitarian choice. In Experiment 1, we gauged the decision in five such points, for equal risks to driver and pedestrians. In Experiment 2, we gauged the decision in nine points, for mixed risks. In this third experiment, we wanted to gauge what was the decision function in this space, specifying when people make the utilitarian choice. To accomplish this, participants were asked to decide, for five different levels of risk to the driver, at what level of risk to the pedestrians would they switch from driving towards the pedestrians to driving towards the wall. The experiment followed a repeated measure 5-level design: *risk of injury to driver* (10% vs. 30% vs. 50% vs. 70% vs. 90%). For each level of risk to the driver, participants had to program their AV using one of three options, Figure 4A: 1) always choose to drive towards the pedestrians; 2) always choose to drive towards the wall; and, 3) drive towards the wall but only if the risk of injury to the pedestrians was above a certain threshold which the participant had to specify using a slider; if the risk to the pedestrians was below (or equal to) the threshold, then the car would drive towards the pedestrians.

We recruited a new sample of 111 participants from Amazon Mechanical Turk. We, first, wanted to understand when participants chose to drive towards wall, drive towards the pedestrians, or specify a decision threshold. We ran repeated measure ANOVAs and the results indicated that, as shown in Figure 4B, participants tended to be more likely to always drive towards the wall, the lower the risk to the driver, *F* (4, 448) = 5.67, *p* < .001, and $\eta_{p}^{2}$ = 0.048; tended to be more likely to always drive towards the pedestrians, the higher the risk to the driver, *F* (4, 448) = 4.65, *p* < 0.001, and $\eta_{p}^{2}$ = 0.040; and tended to be more likely to specify a threshold for nonextreme levels of risk to the driver, *F* (4, 448) = 2.10, *p* = .080, and $\eta_{p}^{2}$ = 0.018. We, then, created a unified decision measure for the switch threshold by following this procedure: 1) if the participant specified a threshold, we kept that value; 2) if the participant chose to always drive towards the wall, we set the value to 0; and, 3) if the participant chose to always drive towards the pedestrians, we set the value to 100. We ran a repeated measure ANOVA on this new measure and the results, shown in Figure 4C and detailed in the Supplemental Material, revealed a main effect, *F* (4, 448) = 9.39, *p* < .001, and $\eta_{p}^{2}$ = 0.077. This effect provides clear insight on our research question on the interaction between risk to driver and risk to pedestrians (RQ1), indicating that the switch threshold increased with increased risk to the driver but the threshold was always below the corresponding risk to the driver; i.e., the decision function had a positive slope that was lower than the unit.

### 2.4 Experiment 4

The game-theoretic formulation for the moral dilemma was motivated by the assumption that individuals are influenced by others. In the fourth and last experiment, we wanted to test this assumption. To accomplish this, we devised a manipulation where participants, before making their decision in a scenario, would receive prior information about what was the “common” response in that scenario. This information was presumably derived from similar studies conducted in the past. Additionally, after each scenario, participants would receive feedback about the other (three) drivers’ decisions (in the 4-person prisoner’s dilemma), and these decisions were compatible with the information provided about prior behavior. We compared participant decisions when others tended to make the utilitarian vs. nonutilitarian choice. For each level of this between-participants factor, we asked participants to make a decision in three scenarios where the risk to the driver was always high (90%) and the risk to the pedestrians was, respectively, 10% vs. 50% vs. 90%. The order that the scenarios presented was counterbalanced across participants.

**FIGURE 5:**
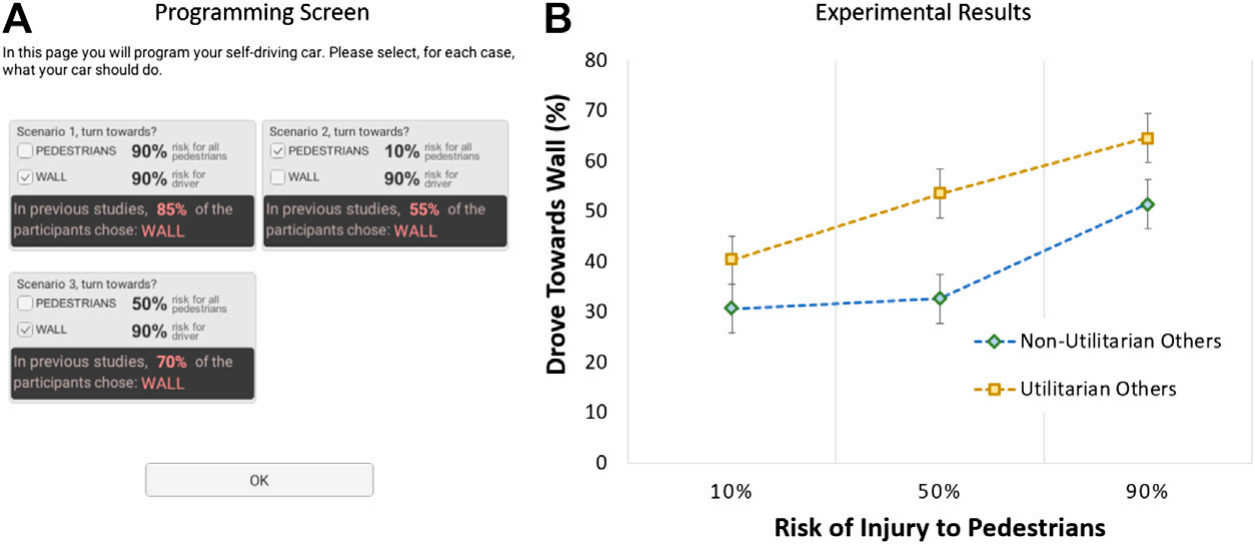
Moral dilemma software and results in Experiment 2: **(A)** programming interface; **(B)** the influence of others’ behavior on utilitarian choice (error bars show standard errors).

The experiment, thus, followed a 2 × three mixed factorial design: others’ behavior (utilitarian vs. nonutilitarian; between-participants) × pedestrians risk (10% vs. 50% vs. 90%; within-participants). For utilitarian others, participants were told that “in previous studies, 55%\70%\85% of the participants chose WALL,” respectively, for a pedestrian risk level of 10%\50%\90%; see Figure 5A. After the scenario was over, participants would also learn that the other three owners always chose to drive towards the wall, except when the risk to pedestrians was 10%, in which case only two drove towards the wall. For nonutilitarian others, participants were told that “in previous studies, 5%\20%\35% of the participants chose WALL,” respectively, for a pedestrian risk level of 10%\50%\90%. Moreover, the other owners would always drive towards the pedestrians, except when the risk to pedestrians was 90%, in which case only two drove towards the pedestrians.

We recruited a new sample of 200 participants from Amazon Mechanical Turk. To analyze the data, we ran a mixed ANOVA on the utilitarian choice rate. The results are shown in Figure 5B and detailed descriptive statistics are shown in the Supplemental Material. The analysis confirmed a main effect of pedestrians risk, *F* (2, 396) = 11.67, *p* < .001, and $\eta_{p}^{2}$ = 0.056: participants were more likely to make the utilitarian choice, the higher the risk to pedestrians. The results also showed a main effect of others’ behavior, *F* (2, 396) = 12.39, *p* = .001, and $\eta_{p}^{2}$ = 0.059: participants were more likely to make the utilitarian choice with utilitarian than nonutilitarian others. These findings, confirming our hypothesis H3, indicating that other people’s behaviors influenced participants’ decisions, suggesting participants adjusted their decisions to be more in line with what others do. Moreover, in line with our hypothesis H4, participants did not strategically converge to the nonutilitarian choice and often made the utilitarian choice, even when engaging with nonutilitarian others.

## 3 General Discussion

As autonomous machines, such as robots and automated vehicles, become pervasive in our daily lives, it will be inevitable that machines will face situations where they must make decisions that risk human life. Giving back control to humans for these split-second decisions is unsatisfactory as machines are capable of processing more information and faster than a human would and, thus, likely to make better decisions. In particular, in addition to perceiving which humans are at risk, machines are also in a position to assess the risk to these humans given advances in technology to perceive the surrounding environment. But which autonomous decisions do humans prefer, given the risk profile of the situation?

We presented experimental evidence that shows that the risk of injury plays a pervasive role in people’s preferences in these moral dilemmas. Our experiments indicate that people were more likely to prefer to save the pedestrians, the higher the risk to pedestrians; moreover, the risk to pedestrians tended not to have to be as high as for the driver to motivate this utilitarian choice. Earlier research showed that when forced to make a decision that led with 100% certainty to the death of the targeted human(s), some participants were still seemingly willing to make the ultimate sacrifice (Swann et al., 2014; Bonnefon et al., 2016; Faulhaber et al., 2019). However, here, we show that this decision is really moderated by the perceived risk of the situation. The results for Experiment 3 reveal a decision function that is mostly linear, with the threshold for switching to the utilitarian choice being higher, the higher the risk to the driver. Nevertheless, in general, people were likely to switch to the utilitarian choice when the risk to the pedestrians was well below the risk to the driver. This result clearly indicates that research on moral dilemmas with AVs should account for this important factor and, moreover, decision-makers need to consider the risk profile of different situations when designing or legislating how AVs should behave.

We explored a game-theoretic formulation for moral dilemmas involving autonomous machines based on the (*n*-person) prisoner’s dilemma. This framework acknowledges that some people adjust their behavior according to what others in society do (Crutchfield, 1955; Kollock, 1998; Hornsey et al., 2003; Kundu and Cummins, 2013; Rand and Nowak, 2013; Bostyn and Roets, 2017; Gogoll and Müller, 2017). These social aspects of moral decision-making are absent, or at least only implicit, in common formulations of AV moral dilemmas (Pan and Slater, 2011; Francis et al., 2016; Awad et al., 2018; Faulhaber et al., 2019; McManus and Rutchick, 2019). This formulation, thus, is likely to have higher ecological validity (Conitzer et al., 2017; Gogoll and Müller, 2017). Accordingly, in our fourth experiment, we report clear evidence that participants adjusted their decisions to match what others did. This is in line with arguments that people conform to the majority (Crutchfield, 1955; Hornsey et al., 2003; Kundu and Cummins, 2013; Bostyn and Roets, 2017) and that people are likely to reciprocate (directly or indirectly) choices that benefit the collective (Dawes, 1980; Kollock, 1998; Rand and Nowak, 2013). The findings, however, did not find that people strategically converge to the nonutilitarian choice, as suggested by Gogoll and Müller (2017). In fact, people often made the utilitarian choice, even when facing nonutilitarian others.

Ultimately, the end user is unlikely to have the opportunity to specify the complete moral policy for their AV without restrictions from the government and manufacturer. Some researchers argue that drivers should have minimal say in the debate (Gogoll and Müller, 2017; Faulhaber et al., 2019), whereas others question if external entities, such as government, should have a strong say (Bonnefon et al., 2016). We acknowledge the importance of understanding the preferences for these different stakeholders—owner, manufacturer, and government—and argue for the scientific study of this important factor in future work.

The findings presented here have important practical implications. Our evidence suggests that, rather than simple rules (e.g., save the pedestrians at all costs), users are engaged in a cost-benefit analysis involving the relative risk of injury to all involved parties (Bennis et al., 2010). Moreover, as illustrated in Experiment 4, decisions are influenced by local social norms (i.e., the choices made by other drivers). Thus, different segments of the population or different cultures may weigh costs and benefits differently, and these decisions may be susceptible to media campaigns about appropriate social norms. Manufacturers, therefore, should consider providing some measure of control to the owner, at least within a range permitted by government and manufacturer restrictions, to encourage the adoption of AV technology. Additionally, this research clearly notes that owners care about the risk profile in different situations and, thus, AV technology should support risk assessment and transparency to facilitate more nuanced decisions in moral dilemmas. Our results suggest that safety features that protect pedestrians may carry more weight in the decision to purchase an AV than in a regular car.

The work presented here has some limitations that introduce opportunities for future study. We mentioned above the importance of comparing decisions by different stakeholders (e.g., owner vs. manufacturer vs. government), but it should also be relevant to compare decisions in the role of AV owner vs. pedestrians. Adopting the other side's perspective can increase cooperation in social dilemmas (de Melo et al., 2019) and, in this case, may lead to an effect on utilitarian choice. Just as importantly, the source of the risk can be manipulated—e.g., why are the pedestrians crossing the street? Are they being careless?—as that is likely to influence decision-making and, simultaneously, those kinds of contextual inferences are likely to be supported by AV technology. Other researchers have also noted that other physical characteristics—e.g., age of involved parties—may influence preferences in these dilemmas (Awad et al., 2018; Faulhaber et al., 2019), and those factors should also be studied in a game-theoretical formulation of AV moral dilemmas. Varying the number of pedestrians should also influence the risk calculations and, thus, impact people’s decisions (Faulhaber et al., 2019). In the current design, the moral dilemma is presented as a mixture of text and video, but it has been noted that mode of presentation can influence the decision (Francis et al., 2016) and, thus, is another potentially relevant factor to study. Overall, these questions emphasize the importance of the kind of experimental work presented here, as it has the potential to shed light on people’s preferences about moral behavior in machines, inform the design of autonomous machines people are likely to trust and adopt, and, perhaps, even introduce an opportunity to promote a more moral society.

## Data Availability

The original contributions presented in the study are included in the article/Supplementary Material; the participant data for all experiments is included in the Supplementary Material; further inquiries can be directed to the corresponding author.

## Footnotes

1 Although we choose these terms to maintain consistency with prior research on AV moral dilemmas, the term “utilitarian choice” may be seen as problematic when we consider risk. Swerving to avoid pedestrians maximizes collective welfare when death is certain, but a utilitarian might conclude, when risk of injury to pedestrians is quite low that welfare could be maximized by protecting the driver.

2 In exploratory fashion, we also manipulated, between-participants, whether the moral dilemma was associated with an explicit payoff matrix. However, this factor did not interact with risk of injury and, so, we did not focus on it for the remaining experiments, where we did not provide a payoff matrix. However, see the Supplemental Materials for more details on these results.

3 In exploratory fashion, we also manipulated, between-participants, the role of the participant (AV owner vs. manufacturer) and others' behavior (utilitarian vs. nonutilitarian vs. neutral). However, these factors did not produce significant effects. We leave the study of the role of the participant for future work; see the General Discussion for more remarks on this. Regarding others' decision, we followed up with a better and more focused design—Experiment 4—to study this important factor. Please see the Supplemental Materials for more details on these preliminary results for this experiment.
