# Supplementary material for: Risk of Injury in Moral Dilemmas With Autonomous Vehicles
Source: Front Robot AI. 2021 Jan 20;7:572529. doi: 10.3389/frobt.2020.572529 (PMC8239464; doi:10.3389/frobt.2020.572529)
Supplement: Supplementary file 6 [file DataSheet1.PDF]

Supplementary Material

1 SUPPLEMENTARY APPENDIX

1.1 Sample size, demographics, and financial incentives

In this section we describe the sample size calculation, sample demographics, and financial incentives for all experiments. We always recruited participants from Amazon Mechanical Turk and we only sampled participants from the United States with an excellent performance history (95% approval rate on previous tasks). To estimate sample sizes, we followed the power calculations proposed by Cohen[1] and implemented in G*Power[2] – a software that is often used by behavioral researchers.

For Experiment 1, we started by predicting a medium effect size (η_p^2 = 0.045, Cohen's f = 0.22) for the between-participants effects. Thus, for α = .05 and statistical power of .80, the recommended total sample size was 102 participants. In practice, because some participants did not complete the task correctly or in the allotted time, we only recruited 94 participants ($\sim$47 participants per condition). Regarding demographics, 64.9% of the participants were males and the age distribution was as follows: 22 to 34 years, 57.4%; 35 to 44 years, 26.6%; 45 to 54 years, 10.6%; 55 to 64 years, 4.3%, over 64 years, 1.1%. Participants were paid $2.00 for participation.

For Experiment 2, building on the results for the previous experiment, we predicted a small effect size (η_p^2 = 0.025, Cohen's f = 0.16) for the between-participants effects, and for α = .05, statistical power of .80, the recommended total sample size was 288 participants; in practice, we recruited 276 participants. Regarding demographics, 62.1% of the participants were males and the age distribution was as follows: 18 to 21 years, 1.1 %; 22 to 34 years, 53.5%; 35 to 44 years, 27.3%; 45 to 54 years, 11.0%; 55 to 64 years, 6.0%, over 64 years, 1.1%. Participants were paid $2.00 for participation.

For Experiment 3, we wanted to increase the power in our sample to increase the chances of picking up any effect due to differences in risk, as well as differences in pairwise comparisons for various risk levels. So, we predicted a small effect size (η_p^2 = 0.02, Cohen's f = 0.14) for the within-participants effect, and for α = .05, statistical power of .95, the recommended total sample size was 93 participants; in practice, we recruited 111 participants. Regarding demographics, 63.1% of the participants were males and the age distribution was as follows: 18 to 21 years, 1.8 %; 22 to 34 years, 48.6%; 35 to 44 years, 27.0%; 45 to 54 years, 7.2%; 55 to 64 years, 10.8%; over 64 years, 3.6%; and, declined to answer, 0.9%. We adjusted the financial incentive to average values at the time of the experiment, and raised the participation fee to $2.75.

For Experiment 4, to estimate the sample size we focused on the between-participants effect for other's behavior. We predicted a small to medium effect size (η_p^2 = 0.04, Cohen's f = 0.20) for the between-participants effect, and for α = .05, statistical power of .95, the recommended total sample size was 210 participants; in practice, we recruited 200 participants, since some participants did not complete the task correctly or in the appropriate amount of time. Regarding demographics, 63.1% of the participants were males and the age distribution was as follows: 18 to 21 years, 0.5 %; 22 to 34 years, 48.5%; 35 to 44 years, 30.5%; 45 to 54 years, 14.5%; 55 to 64 years, 3.5%; and, over 64 years, 2.5%. Participants were paid $2.75 for participation.

[1] Cohen, J. Statistical power analysis for the behavioral sciences, 2nd edn (Lawrence Erlbaum Associates, 1988).

[2] Faul, F., Erdfelder, E., Lang, A. Buchner, A. G*Power 3: A flexible statistical power analysis program for the social, behavioral, and biomedical sciences. Behav. Res. Method. 39, 175-191 (2007).

1.2 Extended Analyses for Experiment 1

The experiment followed a 5×2 mixed factorial design: *risk of injury* (10% vs. 30% vs. 50% vs. 70% vs. 90%; within-participants) × *payoff matrix* (no payoff vs. payoff; between-participants). The risk of injury factor was the focus of the experiment and was motivated in the main text. Regarding the payoff matrix, approximately half of the participants engaged in this dilemma with a formal payoff matrix, as shown in Figure 1B in the main text. These participants were told that their odds of winning a $30 lottery would increase with their performance, as defined by the payoff matrix. The other half of participants were not given any payoff matrix and were simply told to imagine themselves facing the moral dilemma. To be fair, they were also allowed to enter the $30 lottery with equal odds for all participants in this condition.

To analyze the data, we ran a risk of injury × payoff matrix mixed ANOVA on utilitarian choice rate. The results showed a main effect of risk of injury, $F(4, 368) = 4.497$, $p = .001$, $\eta_p^2 = .047$, with people being more likely to swerve towards the wall, the lower the risk of injury to the driver and pedestrians. The results also showed a main effect of payoff matrix, $F(1, 92) = 4.317$, $p = .001$, $\eta_p^2 = .045$, with people being less likely to swerve towards the wall if a payoff matrix was given. It is not clear, though, whether this effect happened because people were taking the task more seriously, they were simply distracted by the short-term monetary reward in the experiment, or associating money with choices involving human life fundamentally alters decision making. Nevertheless, the results also showed no statistically significant risk of injury × payoff matrix interaction, $F(4, 368) = 0.522$, $p = .720$, $\eta_p^2 = .006$, which suggests that the effects of risk of injury and payoff matrix were independent of each other. Consequently, given that our focus is on risk of injury, moving forward, we dropped the payoff matrix.

1.3 Extended Analyses for Experiment 2

The experiment followed a 3×3×3×2 mixed factorial design: *risk of injury for driver* (10% vs. 50% vs. 90%; within-participants) × *risk of injury for pedestrians* (10% vs. 50% vs. 90%; within-participants) × *others' behavior* (utilitarian vs. non-utilitarian vs. neutral; between-participants) × *role* (owner vs. manufacturer; between-participants). The risk of injury factors were the focus of the experiment and were motivated in the main text. Regarding role, we wanted to explore whether making the decision in different roles could have an effect, so participants engaged in the task either as the owner or manufacturer of the AV. Prior to programming the AV, participants would be informed about their role and this was reinforced throughout the task. Regarding others' behavior, the idea was to understand if participant's decisions are influenced by what others do and, so, other's behavior depended on the experimental condition: *utilitarian* - others tended to save the pedestrians (in five rounds, two out of three drove towards the wall; in four rounds, all drove towards the wall); *non-utilitarian* - others tended to save themselves (in five rounds, two out of three drove towards pedestrians; in four rounds, all drove towards pedestrians); and, *neutral* - others were just as likely to make the utilitarian as non-utilitarian choice.

To analyze the data, we ran a risk of injury to driver × risk of injury to pedestrians × others' behavior × role mixed ANOVA on utilitarian choice rate. The results showed a main effect of risk to driver, $F(2, 1104) = 141.66$, $p < .001$, $\eta_p^2 = .339$: people were more likely to make the utilitarian choice, the lower the risk for the driver. The results also showed a main effect of risk to pedestrians, $F(2, 1104) = 107.65$, $p < .001$, $\eta_p^2 = .281$: people were more likely to make the utilitarian choice, the higher the risk to pedestrians. There was a statistically significant risk to driver × risk to pedestrians interaction, $F(4, 1104) = 22.73$, $p < .001$, $\eta_p^2 = .076$, which was discussed in the main text. We did not find, however, a main effect of others' behavior, $F(2, 276) = 0.91$, $p = .402$, $\eta_p^2 = .007$, suggesting the current design was not able to pick up this effect; however, a better and more focused design to study this was presented in the main text (Experiment 4).

Table S1. Utilitarian choice and standard errors in Experiment 1.

Risk of Injury (Driver and Pedestrians)	No Payoff		Payoff	
	Mean	*SE*	*Mean*	*SE*
10%	73.91	6.99	56.25	6.84
30%	60.87	7.33	56.25	7.18
50%	65.22	7.27	54.17	7.11
70%	54.35	7.22	33.33	7.07
90%	52.17	7.37	39.58	7.21

Table S2. Utilitarian choice and standard errors in Experiment 2.

Other's Behavior	Risk of Injury (Driver vs. Pedestrians)	Owner		Manufacturer	
		Mean	*SE*	*Mean*	*SE*
Non-Utilitarian	10% vs. 10%	70.00	6.76	71.43	6.83
	10% vs. 50%	86.00	6.01	77.55	6.07
	10% vs. 90%	82.00	6.10	81.63	6.17
	50% vs. 10%	18.00	6.34	20.41	6.40
	50% vs. 50%	58.00	6.96	65.31	7.03
	50% vs. 90%	62.00	6.28	77.55	6.34
	90% vs. 10%	16.00	5.61	16.33	5.67
	90% vs. 50%	28.00	6.57	28.57	6.63
	90% vs. 90%	52.00	6.97	61.22	7.04
Utilitarian	10% vs. 10%	63.83	6.98	65.38	6.63
	10% vs. 50%	74.47	6.20	71.15	5.89
	10% vs. 90%	76.60	6.30	69.23	5.99
	50% vs. 10%	42.55	6.54	32.69	6.21
	50% vs. 50%	53.19	7.18	65.38	6.82
	50% vs. 90%	78.72	6.48	76.92	6.16
	90% vs. 10%	21.28	5.78	26.92	5.50
	90% vs. 50%	34.04	6.77	32.69	6.44
	90% vs. 90%	61.70	7.19	61.54	6.83
Neutral	10% vs. 10%	64.44	7.13	56.52	7.05
	10% vs. 50%	80.00	6.33	69.57	6.27
	10% vs. 90%	80.00	6.43	56.52	6.36
	50% vs. 10%	26.67	6.68	30.43	6.61
	50% vs. 50%	64.44	7.34	47.83	7.26
	50% vs. 90%	80.00	6.62	58.70	6.55
	90% vs. 10%	22.22	5.91	13.04	5.85
	90% vs. 50%	31.11	6.92	28.26	6.85
	90% vs. 90%	66.67	7.34	41.30	7.26

Finally, we did not find an effect of role, $F(2, 276) = 1.12$, $p = .290$, $\eta_p^2 = .004$, suggesting that our current exploratory design was not sufficient to pick up any differences due to role.

2 SUPPLEMENTARY TABLES

This section shows the means and standard errors for the main measures for Experiment 1 (Table S1), Experiment 2 (Table S2), Experiment 3 (Table S3), and Experiment 4 (Table S4).

Table S3. Threshold for pedestrians risk at which participants switch to utilitarian choice and standard errors in Experiment 3.

Risk of Injury to Pedestrians (Risk to Driver: 90%)	*Mean*	*SE*
10%	27.12	3.59
30%	33.47	3.67
50%	39.42	3.68
70%	45.28	3.86
90%	54.40	4.07

Table S4. Utilitarian choice and standard errors in Experiment 4.

Risk of Injury	Non-Utilitarian Others		Utilitarian Others	
(Driver vs. Pedestrians)	*Mean*	*SE*	*Mean*	*SE*
90% vs. 10%	30.69	4.76	40.40	4.81
90% vs. 50%	32.67	4.84	53.54	4.89
90% vs. 90%	51.49	4.89	64.65	4.94

3 OTHER MATERIALS

The Supplemental Materials also include the experimental data for all experiments and a video of the software used in all experiments.
